# The Effect of Lumen-Apposing Metal Stent (LAMS) in Acute Cholecystitis Unfit for Surgery: Good Tidings

**DOI:** 10.3390/diagnostics15222835

**Published:** 2025-11-09

**Authors:** Valentina Zadro, Giulia Bertoncini, Giuliano Francesco Bonura, Pablo Cortegoso Valdivia, Noemi Gualandi, Paola Soriani, Tommaso Gabbani, Mauro Manno

**Affiliations:** 1 Unit of Gastroenterology and Digestive Endoscopy, Azienda USL di Modena, 41012 Carpi, Italy; v.zadro@ausl.mo.it (V.Z.); gi.bertoncini@ausl.mo.it (G.B.); g.bonura@ausl.mo.it (G.F.B.);; 2 Unit of Gastroenterology and Digestive Endoscopy, Azienda Ospedaliero-Universitaria di Parma,43126 Parma, Italy

**Keywords:** acute cholecystitis, EUS gallbladder drainage, LAMS, unfit for surgery

## Abstract

Acute cholecystitis in patients unfit for surgery presents a significant therapeutic challenge, often requiring alternatives to traditional cholecystectomy. In recent years, endoscopic ultrasound (EUS)-guided gallbladder drainage using Lumen-Apposing Metal Stents (LAMSs) has emerged as a promising minimally invasive approach. This umbrella review synthesizes evidence from existing systematic reviews and meta-analyses evaluating the efficacy and safety of EUS-guided gallbladder drainage with LAMSs in high-surgical-risk patients. The pooled data demonstrate that this approach provides effective symptomatic relief, with high technical and clinical success rates and a low incidence of adverse events. The use of EUS allows real-time visualization and precise access to the gallbladder, contributing to the safety and efficacy of the procedure. These results reinforce the expanding role of endoscopic techniques in managing complex biliary conditions, suggesting that the use of diagnostic EUS in combination with LAMS placement can lead to a significant reduction in the need for surgical intervention among frail patients.

## 1. Introduction

Acute cholecystitis (AC) is a common and potentially life-threatening condition that typically necessitates surgical management [1,2,3,4]. Cholecystectomy remains the gold standard treatment, offering definitive resolution of the disease and preventing recurrence [5,6,7]. However, a subset of patients owing to advanced age, significant frailty, malignancy, multiple comorbidities, or organ failure are considered poor surgical candidates and thus unfit for cholecystectomy [8]. In these cases, alternative minimally invasive options are necessary to achieve gallbladder decompression and control inflammation.

Percutaneous transhepatic gallbladder drainage (PT-GBD) or percutaneous transhepatic cholecystotomy (PTC) has long been the standard of care in non-operative candidates, but it is associated with substantial limitations, including discomfort, risk of infection, dislodgement, and reduced quality of life due to external catheter management. In recent years, EUS-GBD performed with cautery-enhanced lumen-apposing metal stent (LAMS) has demonstrated superiority over PT-GBD in terms of safety, rates of recurrent cholecystitis, and hospital readmissions among patients who are poor candidates for cholecystectomy [9,10]. Therefore, EUS-GBD should be regarded as the preferred method for gallbladder drainage in this high-surgical-risk patients without spontaneous perforation who can tolerate anesthesia and therapeutic endoscopy [11,12,13].

Despite growing interest in LAMSs, data regarding its efficacy, safety, and long-term outcomes in patients unfit for surgery remain fragmented and heterogeneous and there is a need to consolidate current evidence. By synthesizing the existing literature, this systematic review aims to point out the effectiveness and safety of LAMSs in the management of acute cholecystitis in patients deemed unfit for surgery.

### 1.1. Indications for Gallbladder Drainage and Definition of “Unfit for Surgery”

AC is an acute inflammation of the gallbladder, usually due to cystic duct obstruction by gallstones. According to the most recent surgical guidelines [6], diagnosis requires local signs of inflammation (Murphy’s sign or right upper quadrant tenderness) plus systemic signs of inflammation (fever, elevated White Blood Cell (WBC) count or C-Reactive Protein) or imaging findings (gallbladder wall thickening, distension, or pericholecystic fluid). AC is classified as severe (Grade III) when it involves dysfunction of one or more organs, moderate (Grade II) when it is associated with signs of local inflammation, elevated WBC etc., or mild (Grade I) when it does not meet the criteria for moderate or severe disease.

The standard treatment is laparoscopic cholecystectomy. However, this approach is not always feasible due to comorbidities or the patient’s overall clinical condition, which can be stratified using scoring systems such as the Charlson Comorbidity Index (CCI) or the American Society of Anesthesiologists Physical Status Classification System (ASA-PS). Specifically, in cases of moderate-to-severe acute cholecystitis unresponsive to antibiotic therapy, a CCI ≥ 4, an ASA-PS ≥ 3, or the presence of adverse prognostic factors (e.g., serum bilirubin > 2 mg/dL, neurological dysfunction, or respiratory dysfunction) are considered high-risk conditions for cholecystectomy [4]. In these situations, gallbladder drainage (either percutaneous or endoscopic) is recommended as an initial intervention, followed by elective cholecystectomy once the acute phase has resolved. In clinical practice, these assessments are performed jointly by the general surgeon and the anesthesiologist, who evaluate the patient upon hospital admission and again 24–48 h after the initiation of antibiotic therapy.

### 1.2. Technical Aspects

EUS-GBD was initially performed with devices originally designed for other endoluminal procedures, including nasobiliary catheters, double-pigtail plastic stents [14,15,16,17] and self-expandable metal stents [18,19]. However, double-pigtail plastic stents were associated with a higher risk of bile leakage and peritonitis, whereas SEMS carried the risk of migration and contralateral wall injury. To address these limitations, LAMSs—a subclass of antimigratory self-expandable metal stents (SEMSs)—were developed. Although LAMS were first introduced for EUS-guided drainage of peripancreatic fluid collections, the clinical applications have since evolved and now extend to EUS-guided biliary and gallbladder drainage, enteric anastomoses [20,21,22,23], showing higher technical and clinical success rates along with an improved safety profile [19,24,25,26].

Here is the step-by-step description of EUS-GBD using a LAMS:

**Patient Preparation:** The patient must fast for at least 6 h prior to the procedure. Since patients with acute cholecystitis are already receiving antibiotic therapy as part of standard medical management (e.g., piperacillin/tazobactam or a third-generation cephalosporin), no additional prophylactic antibiotics are required. The procedure is performed under moderate to deep sedation or general anesthesia, depending on the patient’s condition, with the patient placed in the left lateral decubitus position.

**EUS Evaluation:** A linear echoendoscope is used to identify the distended gallbladder. There are two preferred access points: the duodenal bulb (for a cholecystoduodenostomy), or gastric antrum (for a cholecystogastrostomy) [27]. Definitive evidence comparing LAMS patency rates between the transgastric and transduodenal approaches is still lacking, but preliminary data indicate that their outcomes are largely similar [28]. The transduodenal approach is generally preferred due to its relative stability, as the duodenum is less affected by peristaltic motion compared to the distal stomach. This route may also reduce the risk of food impaction. Conversely, in patients who may be considered for future cholecystectomy, the transgastric approach may be advantageous, as surgical management of a gastric wall defect is technically more straightforward than repair of a duodenal wall [29,30]. Color-Doppler is used to exclude intervening vascular structures between the GI lumen and the gallbladder and the optimal puncture site is chosen based on proximity and safety.

**Figure 1:**
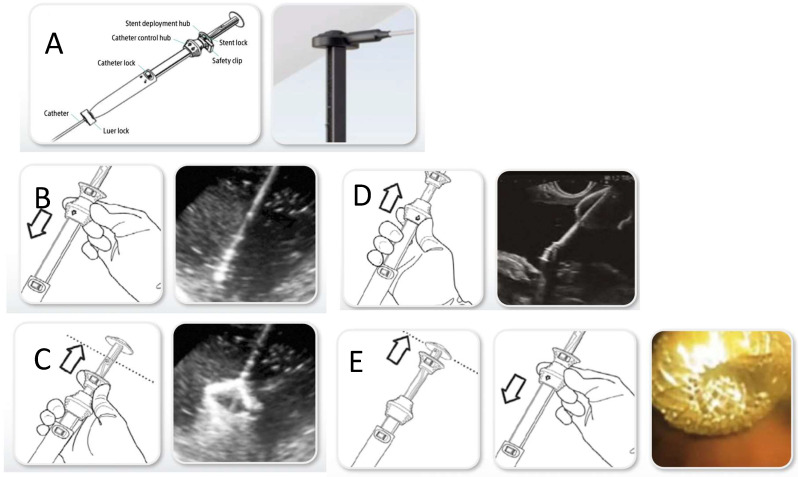
Step-by-step deployment of the Hot-Axios stent. (**A**) The catheter is introduced through the therapeutic channel of the echoendoscope. (**B**) The catheter is advanced into target site (gallbladder lumen) directly using a pure cutting current. (**C**) Deployment of distal flange in the gallbladder. (**D**) The catheter is retracted until the distal flange sits gently against the inner wall. (**E**) Deployment of proximal flange into the gastric or the duodenal lumen. Courtesy of Boston Scientific.

**Figure 2:**
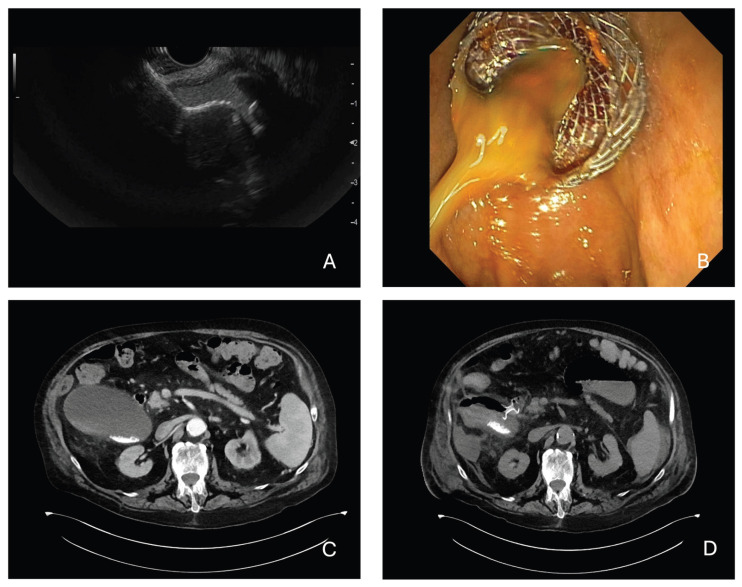
Top Panel (**A**,**B**): EUS-guided gastro-gallbladder drainage with LAMSs. Bottom panel: CT images of a patient with acute calculous cholecystitis unfit for surgery, before (**C**) and after (**D**) LAMS placement between gallbladder and duodenal bulb.

**Gallbladder Access and Stent Deployment**: Two main techniques can be used depending on the equipment and operator preference. The first one is direct Cautery-Assisted (free-hand) Technique. A cautery-enhanced LAMS delivery system (e.g., Hot AXIOS™) is advanced directly into the gallbladder without a guidewire (Figure 1). The electrocautery tip allows direct transmural access under EUS guidance and, once in the gallbladder, the distal flange is deployed under EUS and endoscopic guidance, then the proximal flange is released into the GI lumen creating a secure fistulous tract (Figure 2). The second technique is Wire-Guided: a 19G EUS needle is used to puncture the gallbladder, then bile is aspirated to confirm position and a guidewire (usually 0.025 or 0.035 inch) is advanced and coiled into the gallbladder lumen. The tract is then dilated (e.g., with a cystotome or balloon dilator). A non-cautery LAMS or standard fully covered metal stent can then be deployed over the wire. In both cases, successful deployment results in immediate bile drainage, often visualized endoscopically [31]. For LAMS placement, high-quality comparative evidence between strategies is lacking, and the choice depends on the clinical scenario and operator expertise. Experience from EUS-guided enteric anastomoses warns of technical challenges when a guidewire advances into a mobile target, while direct placement offers the advantage of being performed entirely under EUS/endoscopic control without fluoroscopy. Moreover, in a very recent multicenter retrospective study concerning the technical aspects of performing a choledochoduodenostomy, the use of the guidewire-assisted technique was identified as one of the major risk factors for procedural failure [32]. However, certain rescue maneuvers or additional interventions may still require fluoroscopic guidance, and no consensus exists on the optimal room set-up for EUS-GBD. As a result, EUS-GBD is now most often performed by advancing the Hot-LAMS system directly into the gallbladder, deploying the distal flange under EUS guidance, and then releasing the proximal flange either intra-channel or under direct endoscopic visualization. Nonetheless, the conventional guidewire-assisted technique remains valuable in challenging cases—such as sclerotic or poorly distended gallbladders—where the risk of stent misdeployment is higher and salvage maneuvers may be required [33].

Here is an overview of the different commercially available LAMSs used for endoscopic gallbladder drainage:

- **AXIOS** (Boston Scientific, Marlborough, MA, USA): introduced in 2012 is a fully covered nitinol with a dumbbell (“yo-yo”) shape that prevents migration.
- **Hot-AXIOS** (Boston Scientific, USA) is the most widely used cautery-enhanced LAMS system worldwide and is available in lumen diameters of 6, 8, 10, 15, 20 mm and lengths of 10–15 mm body. FDA-approved in the USA for pancreatic pseudocysts, Walled-Off Necrosis (WON), EUS-GBD in high-risk cholecystitis, and biliary drainage post-ERCP failure.
- **Spaxus** (Taewoong Medical, Goyang-si, South Korea) is a fully covered nitinol LAMS with bilateral flanges; designed for pseudocyst drainage, EUS-GBD, EUS-guided biliary drainage (EUS-BD).
- **Hot-Spaxus** (Taewoong Medical, South Korea): added electrocautery tip to permit single-step deployment; not yet available worldwide (e.g., limited in the USA).
- **Hanaro/Hanarostent** (M.I.Tech, Pyeongtaek-si, South Korea): covered nitinol stent with flanges; provides approximation but may be less effective at full lumen apposition; used in pseudocysts, WON, and EUS-GBD.

Although current evidence from indirect comparisons suggests that both stents (Hot-Axios and Hot-Spaxus) perform similarly in achieving technical and clinical success, Hot Spaxus may be associated with a lower rate of adverse events (in particular bleeding) compared to Hot-Axios, but further prospective, head-to-head studies are needed to confirm these findings and assess their clinical relevance [34]. There are currently no comparative studies evaluating different LAMS sizes. Selection is typically based on the size of the gallstones, and the likelihood of future reinterventions via peroral transluminal cholecystoscopy. For this latter procedure, a LAMS diameter of at least 10 mm—ideally 15 mm—is recommended [33].

Both devices require a linear echoendoscope, a compatible electrosurgical unit and CO_2_ insufflation. The wall-to-wall distance should ideally be <10–15 mm. Fluoroscopy is optional but useful, especially in difficult cases or if guidewire rescue is anticipated.

**Adverse events and mortality rate:** A recent Italian multicenter retrospective study [35] reported the rates of early and late adverse events (AEs) and mortality associated with EUS-GBD with LAMSs in patients with AC who were unfit for surgery. The overall AE rate was 10.3%, with the most frequent complication being intraprocedural LAMS dislodgement (3 of 116 patients, 2.5%), which was managed endoscopically in most cases. The 30-day mortality rate was 19.8%, predominantly related to patients’ underlying conditions, such as advanced malignancies, heart failure, and renal or hepatic impairment. These findings are consistent with results from another international multicenter study [31] and reported in detail in Table 1**.** Regarding late adverse events, it is worth mentioning the rare possibility of a gallstone becoming impacted within the LAMS, requiring its removal through fragmentation (either electrohydraulic or laser lithotripsy) [36].

**Post-Procedure Care**: The patient should be clinically and biochemically monitored to rule out signs of infection, bleeding, or stent-related complications and continue antibiotics based on clinical response. Follow-up imaging may be used to confirm stent position and gallbladder decompression, but they are not required.

### 1.3. Contraindications

EUS-GBD is contraindicated in the presence of gallbladder perforation, biliary peritonitis, large-volume ascites, significant coagulopathy, or anesthesia intolerance. Surgical consultation is advised to assess future cholecystectomy feasibility and the desirability of preserving normal anatomy, as achieved with Endoscopic Transpapillary Gallbladder Drainage (ETP-GBD), since EUS-GBD necessitates fistula repair at surgery [37].

### 1.4. Stent Removal

An important point of discussion in the management of EUS-GBD with LAMS is whether these devices should be removed electively or left in place. The available evidence is heterogeneous, and no universally accepted recommendation exists.

Most centers agree that very early removal should be avoided, as studies have shown that removing the stent before four weeks is associated with lower rates of clinical success. The maturation of the cholecystoenteric tract generally requires at least four weeks, and removal prior to this interval may increase the risk of recurrence or technical failure. For this reason, many experts advocate scheduled removal between four and eight weeks after placement, once the fistulous tract is consolidated. In some cases, particularly when gallstones or sludge are still present, the LAMS is removed and replaced with plastic double-pigtail stents to maintain long-term drainage while reducing the risk of late complications [38,39,40,41].

On the other hand, several recent series have reported favorable outcomes with intentional long-term indwelling of LAMS, especially in patients who remain permanently unfit for surgery or have limited life expectancy. In these patients, long-term stent patency has been associated with acceptable rates of adverse events and low recurrence of cholecystitis. Nonetheless, prolonged indwell rarely predisposes to delayed complications such as stent occlusion, food impaction, buried stent, or migration. The risk could be influenced by the route of access, with transduodenal placement associated with fewer problems compared to transgastric access [42].

In the setting of interval cholecystectomy, removal of the LAMS before surgery is usually advised to avoid technical difficulties related to the fistulous tract [43]. For surgical candidates with normal life expectancy but unfit for immediate surgery, it is advisable to discuss the case in advance with the surgeons. The placement of a LAMS is not an absolute contraindication to cholecystectomy; however, it may increase the technical complexity of the procedure due to potential wall defects or adhesions resulting from the LAMS deployment, requiring open cholecystectomy rather than laparoscopic [44,45].

Overall, the decision regarding stent removal should be individualized, considering patient comorbidities, life expectancy, the anticipated need for future surgery, and the local expertise of the treating center. Current guidelines recognize the lack of a standardized approach and support a tailored strategy balancing the benefits of elective removal with the potential safety of long-term indwelling in selected cases [46,47,48].

### 1.5. Comparison with PT-GBD and ETP-GBD

The three modalities of gallbladder drainage have their respective advantages and disadvantages [49]. PT-GBD and ETP-GBD tended to be performed in patients who still had the potential to undergo laparoscopic cholecystectomy [50]. ETP-GBD is associated with lowest rates of reintervention, unplanned admissions and mortality. PT-GBD, despite its high clinical success, is associated with the highest rates of subsequent interventions and unintended hospitalizations due to the most common adverse events like infections, catheter clogging or migration and AC recurrence [51,52] (Table 2). On the other hand, EUS-GBD is preferred in patients who are not planned to undergo cholecystectomy because of poor premorbid status [53]. It is associated with higher rates of clinical success and with lower rates of recurrent episodes of cholecystitis but, although EUS-GBD with cautery-enhanced LAMS has demonstrated superior outcomes in terms of safety, reintervention and readmission rates [54,55,56], PT-GBD remains the most widely used modality due to broader availability and lower cost [1].

Although EUS-GBD shows excellent outcomes and may be the most effective minimally invasive approach for acute cholecystitis, no single drainage strategy is universally superior. Endoscopic drainage (ETP-GBD or EUS-GBD) requires patients fit for sedation; in unstable patients, PT-GBD is the only feasible option, as it can be performed with minimal or no sedation under local anesthesia, and it is preferred in gallbladder perforation. In operable patients, PT-GBD or ETP-GBD may be favored when future cholecystectomy is possible, as they preserve anatomy and facilitate surgery; ETP-GBD is also suitable when ERCP is indicated or in patients with ascites, coagulopathy, or anticoagulant use, as it avoids sphincterotomy. Conversely, EUS-GBD is the only endoscopic option when the papilla or cystic duct is inaccessible (e.g., altered anatomy, obstruction) and uniquely allows for direct gallbladder interventions, making it preferable if stone removal or other therapeutic procedures are anticipated [33].

### 1.6. Technical and Operational Requirements

Training for EUS-guided drainage procedures requires further investigation. An international multicenter retrospective study by Teoh et al. published in 2019 found that endoscopists with fewer than 25 EUS-GBD procedures had a higher risk of unplanned procedural events and adverse outcomes within 30 days [31]. The retrospective design of the study introduces potential selection and lead-time biases. Furthermore, outcome comparisons across different stent systems should be approached with caution [31]. More research is necessary to define optimal training methods and competency assessment. Currently, training mainly relies on unstructured experiences involving simulators, animal models, and mentorship at specialized high-volume centers.

## 2. Materials and Methods

An umbrella review of the literature was conducted to evaluate the efficacy and safety of lumen-apposing metal stents (LAMSs) for the management of acute cholecystitis in patients unfit for surgery.

A comprehensive search was performed in PubMed/MEDLINE, up to 2024. The following keywords were used in various Boolean combinations: “EUS-guided gallbladder drainage”, “gallbladder drainage”, “endoscopic gallbladder drainage”.

Only systematic reviews and meta-analyses published within the last five years were considered eligible for inclusion. Additional eligibility criteria were:

- **Population:** Adult patients with a diagnosis of acute cholecystitis at high surgical risk or deemed unfit for cholecystectomy;
- **Intervention:** Endoscopic ultrasound-guided gallbladder drainage using LAMS;
- **Outcomes:** Technical and clinical success rates, procedure-related adverse events, procedure-related mortality, and need for reintervention or additional procedures.

The exclusion criteria were:

- non-English language studies;
- single case reports or case series with fewer than three patients;
- narrative reviews, editorials, or letters without original patient data;
- meta-analyses that did not clearly specify the type of stent used for transmural EUS-guided drainage (LAMS, fully covered self-expandable metal stents [FC-SEMS], or other) [53,57].

Study selection was performed independently by two reviewers. Discrepancies were resolved by consensus or by consulting a third reviewer. Data extracted included demographic characteristics, indications for drainage, technical aspects of LAMS placement, clinical outcomes, and follow-up.

For pooled analyses, we report whether he included systematic reviews and meta-analyses performed sensitivity analyses, assessed study quality—for example, using the Newcastle–Ottawa Scale (NOS), the Risk of Bias in Non-Randomized Studies of Interventions (ROBINS-I) and the Risk of Bias 2 (RoB 2) tool—or excluded high-risk studies.

## 3. Results

In the included studies, EUS-GBD with LAMS—either analyzed exclusively or as part of a subgroup analysis—was compared with other drainage techniques commonly used in high-risk surgical candidates, namely ETP-GBD or PT-GBD. Specifically, two meta-analyses compared EUS-guided drainage with ETP-GBD, and three compared it with PT-GBD. Among the latter, only one meta-analysis focused primarily on the safety profile of EUS-GBD, reporting exclusively on adverse events, readmission, and reintervention rates [54]. Regarding comparative studies of EUS-GBD vs. PT-GBD, meta-analyses summarized pooled outcomes and generally reported formal quality assessments. However, in one the meta-analysis [13], the included studies were predominantly non-randomized, and only 2 out of 11 were judged to have a low risk of selection bias. Conversely, the meta-analysis by Hayat et al. [54] included only high-quality studies (NOS or RoB 2 score > 5/8). Similarly, the meta-analysis by Luk et al. [58] incorporated one randomized trial with a low risk of selection bias (RoB 2) and four high-quality comparative cohorts (NOS). The key results of the included meta-analyses are summarized in Table 3.

Patient characteristics, such as mean age (±standard deviation), risk classification, and disease severity are summarized in Table 4.

### 3.1. Technical Success

Technical success rates of LAMS placement for gallbladder drainage were comparable to those of percutaneous drainage. In the meta-analysis by Hemerly et al. [13], technical success was achieved in 95.9% of patients in the LAMS group and in 99.6% in the percutaneous drainage group, with no statistically significant difference. Similarly, a second meta-analysis [58] reported, in a subgroup analysis including three studies in the LAMS group, technical success rates comparable to those observed with PT-GBD (OR 0.21, 95% CI 0.04–1.10).

In contrast, EUS-GBD with LAMS demonstrated significantly higher technical success rates compared with ETP-GBD in both analyzed meta-analyses: OR 3.04 (95% CI 1.29–7.15) [59] and 94.7% (95% CI 91.54–96.67) for the LAMS group vs. 87.33% (95% CI 84.42–89.77) for the overall endoscopic gallbladder drainage group [60].

### 3.2. Clinical Success

Clinical success—defined as complete resolution of symptoms as fever/pain and reduction in white cell blood count following gallbladder drainage—was similar between EUS-GBD with LAMS and PT-GBD (OR 1.43, 95% CI 0.42–4.81; 91% in the LAMS group vs. 94.8% in the PT-GBD group) [13,58].

However, when compared with ETP-GBD, LAMS drainage was associated with significantly higher clinical success rates: OR 3.20 (95% CI 1.48–6.94), with 92.1% in the LAMS group vs. 84.2% in the transpapillary drainage group.

### 3.3. Adverse Events, Reintervention and Readmission

The most frequently reported adverse events associated with EUS-GBD using LAMS include bleeding, malposition, perforation, and stent occlusion or migration. Nevertheless, compared with PT-GBD, the adverse event rates reported in two analyzed meta-analyses were lower in the LAMS group (19% vs. PTC 45%, OR 0.43, 95% CI 0.30–0.61 95% CI) [13,54]. In the other one [58], the adverse event rates were similar between the two groups (OR 0.42, 95% CI 0.14–1.28).

When compared with ETP-GBD, one meta-analysis found slightly higher (but not statistically significant) adverse event rates in the LAMS group (11.7% vs. 11.4% for LAMS and ERCP group respectively overall ERCP + EUS-guided-endoscopic drainage group) [60], whereas the meta-analysis by Krishnamoorthi [59] reported comparable rates between the two approaches (OR 1.73, 95% CI 0.84–3.56), although LAMS drainage was associated with a lower incidence of recurrent cholecystitis.

In comparison with PT-GBD, all meta-analyses reviewed reported a lower reintervention rate for EUS-GBD using LAMS. Specifically, in Hemerly’s meta-analysis [13], the reintervention rate in the LAMS group was 1.7% vs. 35% in the PT-GBD group. Similarly, Luk [58] reported an odds ratio (OR) of 0.15 (95% CI, 0.02–0.98) in favor of the LAMS group, findings that were essentially consistent with those observed in Hayat’s study (OR, 0.10; 95% CI, 0.05–0.18) [54]. Compared to ETP-GBD, the LAMS group showed a reduction in the reintervention rate (7.7% vs. 11.4%) [56].

The readmission rate was calculated only in meta-analyses comparing with PT-GBD [13,54,58], and in all three analyses, it was significantly lower in the LAMS group (7.3% vs. 37%; OR 0.14, 95% CI 0.03–0.7; OR 0.18, 95% CI 0.09–0.36).

## 4. Discussion

### 4.1. Benefits and Outcomes: Point of View of the Clinician/Endoscopist

EUS-GBD with LAMS streamlines the pathway into a single, endoscopist-controlled session [61]. The cautery-enhanced, single-step LAMS platform streamlines the procedure under endoscopist control (fewer wire/catheter exchanges) and can be performed as a single endoscopic session, reducing the procedure time [41] and without the logistics of interventional radiology [47]. Compared with PT-GBD and ETP-GBD, meta-analyses show higher technical/clinical success and fewer unplanned reinterventions/readmissions with LAMS-based EUS-GBD than with PT-GBD. Because drainage is internal, scheduling care external tube no longer depends on interventional radiology, simplifying outpatient management, logistics and bed management [62]. The LAMS tract provides stable transluminal access for planned re-entry (e.g., cholecystoscopy, lavage, stone clearance), enabling standardized endoscopic follow-up pathways [41,47]. Operationally, programs report shorter hospital stays with EUS-GBD compared with PT-GBD when expertise and protocols are in place [54,58]. Teams should, however, be trained to recognize and manage LAMS-specific adverse events (e.g., occlusion, migration, buried flange), and establish local protocols accordingly [63].

On top of what has been mentioned, even in meta-analyses including high-quality studies or RCTs, there is a possible patient selection bias in the EUS-GBD group (e.g., better clinical conditions at baseline than patients undergoing PT-GBD) that cannot be fully excluded. Therefore, the observed advantages of EUS-GBD should be interpreted as associations rather than definitive proof of superiority. Prospective randomized trials or multicenter registries with standardized selection criteria are needed to confirm causality.

### 4.2. Benefits and Outcomes: Point of View of Surgeon

Laparoscopic cholecystectomy remains the gold standard treatment for acute cholecystitis in patients who are suitable candidates for surgery [1].

Nonetheless, conversion to open laparotomy occurs in approximately 4.8–8.1% of cases. Such conversions are associated with a substantial increase in healthcare costs (an estimated additional $23,000 per procedure), longer hospital stays, higher rates of adverse events, and an increased risk of 30-day readmission and mortality.

Well-recognized risk factors for conversion include obesity (BMI > 30 kg/m^2^), a history of previous abdominal surgery, impacted gallstones in the gallbladder infundibulum, evidence of choledocholithiasis, and thickening of the gallbladder wall [64].

A retrospective study using propensity score matching compared outcomes of EUS-GBD with LAMS in 30 high-surgical-risk patients with those of 30 lower-risk patients who underwent laparoscopic cholecystectomy. Clinical and technical success rates, as well as adverse event, mortality, readmission, and reintervention rates, were comparable between the two groups. Based on these findings, the authors suggested that an additional subset of patients could be considered for this minimally invasive approach [38].

### 4.3. Benefits and Outcomes: Point of View of Patients

Patients undergoing EUS-GBD are typically frail individuals with multiple comorbidities, in whom laparoscopic cholecystectomy would carry a substantial risk of morbidity and mortality. Traditionally, patients deemed temporarily unfit for surgery would first undergo percutaneous drainage; once their clinical condition improved, laparoscopic cholecystectomy was performed to reduce the incidence of biliary-related complications such as cholangitis, choledocholithiasis, hepatic abscesses, and biliary colic [62].

Several studies have explored the option of leaving LAMS in place long term after drainage, particularly in very elderly patients with limited life expectancy. In a recent multicenter USA study [65], the rates of adverse events, recurrent acute cholecystitis, and long-term outcomes were similar whether the LAMS was left in situ or removed after a median of 38 days (IQR 32–61). In another prospective study including 45 patients who completed 1-year follow-up with LAMS in situ [46], the 1-year risk of AEs and of severe AEs was 18.8% (11–31.2%) and 7.9% (3.3–18.2%, respectively, showing that long-term LAMS indwell does not increase the risk of delayed AEs following EUS-GBD. Moreover, it was found that the only risk factor for recurrent biliary events was pancreatobiliary malignancy (33% vs. 1.5%, *p* = 0.001).

These findings suggest that EUS-GBD with LAMS can be considered a definitive, lifelong solution in selected patients. This is due to its anti-migration design, larger lumen diameter—which promotes more effective drainage—and the absence of an external tube, as required in percutaneous drainage. The latter not only improves patient comfort but also reduces the risk of tube dislodgement or infection [66].

## 5. Conclusions

EUS-GBD with LAMS represents an effective and minimally invasive alternative for managing acute cholecystitis in patients unfit for surgery. Current evidence shows comparable technical and clinical success rates to percutaneous drainage, with fewer adverse events, lower reintervention and readmission rates, and improved patient comfort.

Given its potential to streamline care and enable endoscopic reinterventions, EUS-GBD with LAMS may become the preferred therapeutic option in surgical high-risk patients. Further prospective studies are needed to optimize patient selection, stent management, and long-term follow-up strategies.

## Figures and Tables

**Table 1:** AEs and mortality rate in EUS-GBD with LAMS and PT-GBD.

	Early AEs	Late AEs	Mortality Rate (30-Day)
**EUS-GBD with LAMS**	**Dislodgement: 2.5%**	Migration: 0.8%	19.8%
	Bleeding: 1.7%	Perforation: 0.8%	
	Perforation: 0.8%	Buried stent: 0.8%	
	Malpositioning: 0.8%	AC recurrence: 0.8%	
**PT-GBD**	**Dislodgement: 8.6%**	**Infections: 6.2%**	15.4%
		**AC recurrence: 9%**	
		Obstruction: 4–15%	

**Table 2:** Advantages and disadvantages of EUS-GBD with LAMS, PT-GBD and ETP-GBD.

	Clinical Success	Technical Success	AEs	Re-Intervention Rate	External Device	Anesthesia	Hospital LOS	Resource Utilization
**EUS-GBD with LAMS**	High	High	Low	Low	No	Deep sedation, General	Shorter	Endoscopy service, anesthesiologist
**PT-GBD**	High	High	High	High	Yes	Minimal or Local	Prolonged	Interventional Radiology
**ETP-GBD**	Low	Low	High	Low	No	Deep sedation, General	Prolonged	Endoscopy service, anesthesiologist

**Table 3:** Comparison of meta-analysis between EUS-guided drainage with LAMS, PT-GBD and ETP-GBD.

Study (Author, Year)	N. Studies Included	Comparison	Technical Success	Clinical Success	Adverse Event	Reintervention	Readmission
Hemerly et al., 2023 [13]	11 (4 EUS-GBD with LAMS)	EUS-GBD vs. PT-GBD	≈	≈	↓ EUS-GBD with LAMS	↓ EUS-GBD with LAMS	↓ EUS-GBD with LAMS
Luk et al., 2019 [58]	5 (3 EUS-GBD with LAMS)	EUS-GBD vs. PT-GBD	≈	≈	≈	↓ EUS-GBD with LAMS	↓ EUS-GBD with LAMS
Hayat et al., 2024 [54]	6 (all EUS-GBD with LAMS)	EUS-GBD vs. PT-GBD	N/S	N/S	↓ EUS-GBD with LAMS	↓ EUS-GBD with LAMS	↓ EUS-GBD with LAMS
Krishnamoorthi et al., 2020 [59]	5 (3 EUS-GBD with LAMS)	EUS-GBD vs. ETP-GBD	↑ EUS-GBD with LAMS	↑ EUS-GBD with LAMS	≈	N/S	N/S
McCarty et al., 2021 [60]	36 (11 EUS-GBD with LAMS)	Endoscopic GBD (ERCP vs. EUS)	↑ EUS-GBD with LAMS	↑ EUS-GBD with LAMS	≈	↓ EUS-GBD with LAMS	N/S

≈ = similar; ↑ = greater; ↓ = minor; N/S = Not Specified.

**Table 4:** Summary of baseline patient characteristics reported in each meta-analysis.

Study (Author, Year)	Mean Age	Comorbidities	AC Grade (Tokyo Criteria)
Hemerly et al., 2023 [13]	73 ± 12	ASA ≥ III	Grade II/III
		CCI ≥ 4	
Luk et al., 2019 [58]	73.9 ± 7.5	ASA ≥ IV	Grade II/III
		CCI ≥ 4 (mean 5.6)	
Hayat et al., 2024 [54]	73.78 ± 8.01	ASA ≥ III	Grade II/III
		CCI ≥ 4	
Krishnamoorthi et al., 2020 [59]	70.5 ± 8.2	ASA ≥ III	Grade II/III
McCarty et al., 2021 [60]	72.4 ± 5.4	ASA ≥ III	Grade II/III

## Data Availability

No new data were created or analyzed in this study.

## Abbreviations

The following abbreviations are used in this manuscript:

AC	Acute Cholecystitis
AEs	Adverse Event
EUS	Endoscopic Ultrasound
EUS-GBD	Endoscopic Ultrasound Gallbladder Drainage
EUS-BD	Endoscopic Ultrasound Biliary Drainage
ETP-GBD	Endoscopic Transpapillary Gallbladder Drainage
LAMS	Lumen-Apposing Metal Stent
LOS	Length Of Stay
PT-GBD	Percutaneous Transhepatic Gallbladder Drainage
PTC	Percutaneous Transhepatic Cholecystotomy
WON	Walled-Off Necrosis
